# Supplementary material for: A Likelihood Approach for Real-Time Calibration of Stochastic Compartmental Epidemic Models
Source: PLoS Comput Biol. 2017 Jan 17;13(1):e1005257. doi: 10.1371/journal.pcbi.1005257 (PMC5240920; doi:10.1371/journal.pcbi.1005257)
Supplement: S1 File — (TAR.GZ) [file pcbi.1005257.s014.tar.gz › HSPH_Online-SI-Revision/MSS10/n1-mild/n1-mild_NEW2_table-8.pdf]

1	newly 3 weeks spec	14.	23.39	{4., 48.}	98.3571	0.378192	newly 3 weeks cumul	37.	50.25	{17., 90.}	67.3784	0.267198	newly 5 weeks spec	24.	38.86	{6., 76.}	98.0833	0.416706	newly 5 weeks cumul	75.	119.19	{27., 218.}	88.8133	0.319355	newly 63 weeks cumul	382.	681.78	{515., 832.}	88.7958	0.34929
2	newly 3 weeks spec	27.	35.18	{7., 59.}	66.5185	0.350262	newly 3 weeks cumul	86.	110.91	{39., 174.}	54.7791	0.216853	newly 5 weeks spec	28.	27.14	{0., 56.}	59.7143	0.541519	newly 5 weeks cumul	143.	168.98	{48., 274.}	49.5804	0.223338	newly 59 weeks cumul	317.	259.21	{49., 434.}	40.1924	0.268344
3	newly 3 weeks spec	15.	183.26	{35., 306.}	1132.	1.05145	newly 3 weeks cumul	45.	519.08	{90., 829.}	1058.71	0.982597	newly 5 weeks spec	22.	39.73	{1., 95.}	132.864	0.583835	newly 5 weeks cumul	87.	650.02	{145., 914.}	655.448	0.848255	newly 68 weeks cumul	303.	687.88	{181., 924.}	143.815	0.438471
4	newly 3 weeks spec	11.	24.52	{0., 57.}	174.545	0.697806	newly 3 weeks cumul	28.	80.77	{10., 174.}	213.893	0.448443	newly 5 weeks spec	15.	17.83	{0., 43.}	97.5333	0.864326	newly 5 weeks cumul	54.	120.41	{10., 239.}	157.093	0.449399	newly 62 weeks cumul	437.	284.01	{10., 615.}	53.6682	0.549104
5	newly 3 weeks spec	17.	49.6	{1., 100.}	224.471	0.637233	newly 3 weeks cumul	65.	128.61	{19., 222.}	122.538	0.373644	newly 5 weeks spec	31.	42.8	{0., 94.}	99.0968	0.750038	newly 5 weeks cumul	108.	219.82	{34., 404.}	131.963	0.417297	newly 64 weeks cumul	446.	364.63	{34., 785.}	47.9888	0.393188
6	newly 3 weeks spec	1.	24.55	{0., 75.}	2389.	1.02273	newly 3 weeks cumul	6.	41.66	{2., 121.}	622.333	0.592158	newly 5 weeks spec	7.	45.44	{0., 156.}	605.143	0.928417	newly 5 weeks cumul	16.	123.28	{4., 404.}	710.875	0.710708	newly 68 weeks cumul	409.	586.43	{4., 925.}	92.7604	0.657906
7	newly 3 weeks spec	31.	62.81	{0., 137.}	150.806	0.663969	newly 3 weeks cumul	65.	169.82	{23., 412.}	192.554	0.460367	newly 5 weeks spec	13.	50.83	{0., 122.}	343.923	0.976383	newly 5 weeks cumul	100.	277.36	{23., 527.}	211.56	0.547778	newly 67 weeks cumul	468.	444.24	{23., 886.}	55.4444	0.458956
8	newly 3 weeks spec	7.	1.86	{0., 4.}	74.2857	0.926864	newly 3 weeks cumul	20.	5.09	{1., 11.}	74.65	0.763582	newly 5 weeks spec	14.	2.24	{0., 6.}	84.	1.20641	newly 5 weeks cumul	41.	9.28	{1., 21.}	77.3659	0.826195	newly 59 weeks cumul	476.	135.26	{2., 446.}	72.7773	1.16069
9	newly 3 weeks spec	17.	27.55	{0., 60.}	118.059	0.621792	newly 3 weeks cumul	37.	81.31	{14., 147.}	144.243	0.396693	newly 5 weeks spec	20.	18.55	{0., 44.}	77.95	0.849718	newly 5 weeks cumul	77.	122.92	{14., 234.}	95.8701	0.377527	newly 58 weeks cumul	379.	198.55	{14., 366.}	53.3219	0.531857
10	newly 3 weeks spec	34.	34.79	{15., 53.}	33.5	0.175768	newly 3 weeks cumul	83.	91.65	{51., 129.}	29.4578	0.124755	newly 5 weeks spec	34.	39.83	{18., 59.}	40.9706	0.219211	newly 5 weeks cumul	149.	169.41	{90., 243.}	32.0067	0.13559	newly 59 weeks cumul	381.	512.	{246., 705.}	48.3675	0.192267
11	newly 3 weeks spec	5.	3.28	{0., 8.}	76.	0.728745	newly 3 weeks cumul	14.	8.91	{2., 18.}	61.2143	0.472961	newly 5 weeks spec	14.	4.37	{0., 15.}	81.2143	1.22877	newly 5 weeks cumul	35.	17.38	{2., 44.}	70.8571	0.613038	newly 58 weeks cumul	476.	150.96	{3., 562.}	77.4706	1.20763
12	newly 3 weeks spec	4.	7.98	{0., 16.}	155.	0.485553	newly 3 weeks cumul	11.	20.07	{3., 40.}	127.364	0.348221	newly 5 weeks spec	8.	7.89	{0., 18.}	81.125	0.608649	newly 5 weeks cumul	23.	36.29	{6., 68.}	110.13	0.360696	newly 59 weeks cumul	336.	284.97	{6., 596.}	65.2649	0.608874
13	newly 3 weeks spec	29.	25.73	{0., 54.}	65.4138	0.666547	newly 3 weeks cumul	92.	89.82	{19., 160.}	51.2174	0.304125	newly 5 weeks spec	28.	18.75	{0., 45.}	67.75	0.951373	newly 5 weeks cumul	149.	131.85	{19., 244.}	50.2349	0.352219	newly 60 weeks cumul	314.	195.51	{19., 369.}	50.5446	0.470283
14	newly 3 weeks spec	28.	60.92	{29., 94.}	121.143	0.315594	newly 3 weeks cumul	69.	131.27	{73., 196.}	93.8116	0.263889	newly 5 weeks spec	32.	88.09	{49., 116.}	175.344	0.419787	newly 5 weeks cumul	123.	295.73	{162., 420.}	140.431	0.355749	newly 64 weeks cumul	351.	799.04	{676., 890.}	127.789	0.352702
15	newly 3 weeks spec	16.	46.67	{0., 94.}	226.313	0.700151	newly 3 weeks cumul	45.	124.28	{19., 230.}	196.978	0.476849	newly 5 weeks spec	24.	38.88	{0., 85.}	119.75	0.758928	newly 5 weeks cumul	89.	207.59	{22., 355.}	161.697	0.478155	newly 64 weeks cumul	454.	345.29	{22., 752.}	49.3326	0.421374
16	newly 3 weeks spec	21.	37.62	{16., 59.}	86.5714	0.250387	newly 3 weeks cumul	39.	84.07	{44., 126.}	116.487	0.309235	newly 5 weeks spec	16.	55.86	{31., 82.}	249.125	0.514016	newly 5 weeks cumul	78.	187.77	{110., 279.}	140.782	0.355542	newly 62 weeks cumul	411.	735.21	{617., 833.}	78.8832	0.248836
17	newly 3 weeks spec	8.	22.06	{3., 39.}	204.	0.474212	newly 3 weeks cumul	19.	43.85	{9., 76.}	156.579	0.364836	newly 5 weeks spec	11.	44.01	{6., 93.}	317.727	0.61972	newly 5 weeks cumul	37.	121.02	{24., 232.}	243.351	0.476254	newly 65 weeks cumul	358.	753.96	{523., 918.}	120.955	0.417772
18	newly 3 weeks spec	10.	17.21	{0., 39.}	116.7	0.467723	newly 3 weeks cumul	30.	44.55	{13., 95.}	85.3667	0.284592	newly 5 weeks spec	13.	16.86	{0., 51.}	104.769	0.65762	newly 5 weeks cumul	62.	79.51	{17., 183.}	80.3387	0.330796	newly 58 weeks cumul	369.	296.28	{22., 541.}	46.0542	0.426802
19	newly 3 weeks spec	26.	14.05	{0., 31.}	60.8077	0.624914	newly 3 weeks cumul	62.	28.9	{6., 59.}	58.4194	0.499233	newly 5 weeks spec	40.	30.72	{2., 76.}	63.7	0.574326	newly 5 weeks cumul	140.	81.13	{8., 189.}	58.25	0.505272	newly 65 weeks cumul	489.	712.87	{101., 907.}	66.2965	0.377434
20	newly 3 weeks spec	15.	22.82	{0., 57.}	131.2	0.78786	newly 3 weeks cumul	42.	56.12	{7., 144.}	100.095	0.436899	newly 5 weeks spec	12.	25.	{0., 70.}	185.167	0.938744	newly 5 weeks cumul	79.	106.03	{7., 255.}	103.684	0.501936	newly 62 weeks cumul	337.	284.95	{7., 607.}	61.4688	0.603298
21	newly 3 weeks spec	3.	22.82	{0., 61.}	712.	0.97198	newly 3 weeks cumul	24.	83.27	{10., 183.}	267.958	0.534161	newly 5 weeks spec	14.	8.68	{0., 26.}	85.7143	1.20067	newly 5 weeks cumul	48.	106.83	{10., 218.}	156.313	0.451624	newly 62 weeks cumul	267.	117.35	{10., 262.}	59.1498	0.586055
22	newly 3 weeks spec	11.	11.93	{2., 21.}	67.7273	0.372589	newly 3 weeks cumul	21.	25.67	{6., 42.}	66.5238	0.261102	newly 5 weeks spec	8.	22.73	{2., 44.}	210.125	0.521924	newly 5 weeks cumul	39.	64.6	{12., 114.}	97.1282	0.307306	newly 64 weeks cumul	470.	634.53	{76., 863.}	58.9681	0.311994
23	newly 3 weeks spec	17.	32.93	{3., 54.}	116.412	0.446544	newly 3 weeks cumul	40.	92.53	{44., 142.}	141.575	0.381659	newly 5 weeks spec	12.	34.15	{0., 60.}	216.083	0.688938	newly 5 weeks cumul	68.	162.19	{58., 251.}	151.691	0.415291	newly 59 weeks cumul	373.	442.33	{58., 657.}	57.874	0.339543
24	newly 3 weeks spec	11.	15.52	{4., 29.}	76.3636	0.296072	newly 3 weeks cumul	30.	35.79	{13., 60.}	53.9667	0.220294	newly 5 weeks spec	19.	19.39	{1., 39.}	58.5789	0.41431	newly 5 weeks cumul	65.	73.2	{21., 136.}	53.1077	0.246489	newly 61 weeks cumul	445.	437.9	{54., 722.}	50.791	0.380768
25	newly 3 weeks spec	8.	29.46	{15., 43.}	268.25	0.535753	newly 3 weeks cumul	23.	74.36	{45., 100.}	223.304	0.487998	newly 5 weeks spec	6.	34.93	{19., 49.}	482.167	0.734218	newly 5 weeks cumul	43.	141.83	{85., 190.}	229.837	0.498346	newly 58 weeks cumul	330.	561.04	{434., 685.}	70.7455	0.226909
26	newly 3 weeks spec	19.	33.79	{15., 55.}	88.5789	0.250811	newly 3 weeks cumul	55.	80.39	{40., 124.}	60.0545	0.196154	newly 5 weeks spec	16.	46.25	{19., 69.}	190.688	0.425169	newly 5 weeks cumul	94.	168.3	{81., 257.}	86.4043	0.2499	newly 61 weeks cumul	295.	647.98	{505., 778.}	121.105	0.340915
27	newly 3 weeks spec	21.	10.46	{4., 19.}	51.2381	0.388472	newly 3 weeks cumul	42.	24.88	{11., 40.}	42.9048	0.282895	newly 5 weeks spec	24.	15.7	{3., 27.}	45.5833	0.365088	newly 5 weeks cumul	84.	53.58	{19., 85.}	41.7381	0.274977	newly 58 weeks cumul	327.	581.1	{296., 719.}	92.4832	0.331499
28	newly 3 weeks spec	3.	4.42	{0., 11.}	138.667	0.71609	newly 3 weeks cumul	9.	10.84	{2., 25.}	88.4444	0.415144	newly 5 weeks spec	4.	8.01	{0., 25.}	192.25	0.817537	newly 5 weeks cumul	17.	25.01	{2., 60.}	121.	0.470026	newly 64 weeks cumul	356.	345.25	{3., 821.}	87.1826	0.912004
29	newly 3 weeks spec	18.	20.25	{0., 49.}	89.5	0.743904	newly 3 weeks cumul	43.	54.18	{4., 114.}	80.4186	0.402005	newly 5 weeks spec	35.	21.	{0., 52.}	66.0571	1.01894	newly 5 weeks cumul	101.	96.72	{4., 228.}	66.6535	0.485558	newly 61 weeks cumul	354.	308.61	{4., 725.}	70.1554	0.68332
30	newly 3 weeks spec	14.	20.72	{1., 51.}	107.286	0.522365	newly 3 weeks cumul	49.	44.58	{9., 100.}	60.2041	0.341522	newly 5 weeks spec	21.	31.77	{0., 83.}	120.524	0.736573	newly 5 weeks cumul	98.	102.57	{11., 263.}	75.1531	0.412841	newly 65 weeks cumul	412.	468.13	{11., 834.}	74.8325	0.554221
31	newly 3 weeks spec	26.	45.46	{21., 72.}	84.6154	0.247538	newly 3 weeks cumul	72.	104.71	{53., 158.}	57.2083	0.184057	newly 5 weeks spec	44.	63.59	{32., 90.}	54.4318	0.184111	newly 5 weeks cumul	142.	223.59	{112., 329.}	65.1901	0.204336	newly 63 weeks cumul	451.	756.85	{655., 847.}	67.816	0.222218
32	newly 3 weeks spec	6.	10.61	{2., 22.}	119.833	0.36198	newly 3 weeks cumul	17.	23.18	{7., 39.}	76.1176	0.258703	newly 5 weeks spec	8.	15.18	{3., 30.}	124.25	0.388435	newly 5 weeks cumul	37.	51.61	{13., 88.}	82.2973	0.283123	newly 61 weeks cumul	420.	604.16	{51., 799.}	63.5381	0.330976
33	newly 3 weeks spec	16.	18.44	{4., 32.}	59.375	0.33179	newly 3 weeks cumul	37.	43.32	{15., 77.}	52.4324	0.226756	newly 5 weeks spec	21.	25.51	{0., 48.}	69.5714	0.477265	newly 5 weeks cumul	77.	90.73	{25., 158.}	56.7403	0.264852	newly 62 weeks cumul	388.	519.54	{62., 780.}	64.1031	0.348512
34	newly 3 weeks spec	7.	23.42	{0., 61.}	288.	0.763765	newly 3 weeks cumul	27.	61.13	{8., 132.}	158.704	0.407819	newly 5 weeks spec	14.	18.63	{0., 49.}	117.214	0.891966	newly 5 weeks cumul	53.	101.06	{8., 228.}	140.34	0.46277	newly 61 weeks cumul	269.	162.73	{8., 386.}	63.6171	0.629404
35	newly 3 weeks spec	10.	18.21	{4., 35.}	108.3	0.339848	newly 3 weeks cumul	37.	38.25	{14., 68.}	48.5135	0.238856	newly 5 weeks spec	22.	32.79	{7., 69.}	83.1364	0.324802	newly 5 weeks cumul	79.	96.08	{25., 174.}	58.2278	0.239523	newly 63 weeks cumul	462.	711.18	{422., 867.}	64.3117	0.24198
36	newly 3 weeks spec	18.	48.06	{0., 91.}	209.556	0.660542	newly 3 weeks cumul	39.	107.31	{19., 207.}	198.59	0.433965	newly 5 weeks spec	49.	55.55	{0., 122.}	75.7347	0.618751	newly 5 weeks cumul	114.	215.02	{21., 409.}	123.018	0.404466	newly 65 weeks cumul	404.	553.54	{33., 880.}	76.1881	0.402261
37	newly 3 weeks spec	12.	24.4	{0., 67.}	166.5	0.737304	newly 3 weeks cumul	26.	75.31	{13., 169.}	214.962	0.457276	newly 5 weeks spec	13.	16.78	{0., 44.}	121.231	1.06564	newly 5 weeks cumul	55.	112.92	{13., 245.}	146.218	0.450901	newly 61 weeks cumul	310.	195.4	{13., 418.}	57.1806	0.570572
38	newly 3 weeks spec	35.	35.81	{4., 81.}	69.9143	0.474281	newly 3 weeks cumul	65.	79.97	{16., 171.}	72.2	0.324119	newly 5 weeks spec	24.	44.94	{0., 90.}	131.417	0.612109	newly 5 weeks cumul	117.	165.79	{29., 343.}	83.6154	0.358177	newly 65 weeks cumul	464.	520.33	{40., 872.}	57.8556	0.388948
39	newly 3 weeks spec	28.	31.2	{0., 78.}	88.5714	0.797092	newly 3 weeks cumul	72.	102.8	{8., 215.}	92.1389	0.425729	newly 5 weeks spec	31.	18.88	{0., 55.}	72.6452	1.08822	newly 5 weeks cumul	130.	146.78	{8., 316.}	73.0462	0.449967	newly 63 weeks cumul	377.	208.02	{8., 424.}	52.1963	0.575736
40	newly 3 weeks spec	16.	114.89	{43., 186.}	619.313	0.802435	newly 3 weeks cumul	56.	218.71	{85., 366.}	293.232	0.545911	newly 5 weeks spec	15.	131.16	{87., 166.}	775.733	0.924391	newly 5 weeks cumul	83.	486.88	{259., 671.}	487.904	0.74225	newly 66 weeks cumul	397.	851.65	{709., 926.}	115.373	0.329665
41	newly 3 weeks spec	13.	46.32	{3., 106.}	287.077	0.612504	newly 3 weeks cumul	28.	104.81	{13., 224.}	294.321	0.513446	newly 5 weeks spec	36.	62.87	{0., 134.}	119.917	0.626906	newly 5 weeks cumul	89.	224.6	{23., 465.}	179.483	0.465156	newly 67 weeks cumul	337.	607.96	{58., 913.}	108.261	0.433526
42	newly 3 weeks spec	5.	67.57	{18., 134.}	1251.8	1.0205	newly 3 weeks cumul	20.	128.93	{44., 251.}	544.95	0.720618	newly 5 weeks spec	30.	105.01	{30., 152.}	257.7	0.541541	newly 5 weeks cumul	64.	325.39	{120., 545.}	409.484	0.643168	newly 66 weeks cumul	325.	811.76	{594., 923.}	152.591	0.403215
43	newly 3 weeks spec	39.	81.69	{3., 199.}	162.282	0.60863	newly 3 weeks cumul	61.	162.87	{8., 385.}	210.475	0.519625	newly 5 weeks spec	44.	88.97	{2., 174.}	148.023	0.632966	newly 5 weeks cumul	146.	346.85	{27., 737.}	174.61	0.530847	newly 68 weeks cumul	494.	697.83	{208., 922.}	64.9656	0.373567
44	newly 3 weeks spec	19.	16.58	{1., 30.}	52.7368	0.442937	newly 3 weeks cumul	73.	44.64	{13., 68.}	53.4795	0.353413	newly 5 weeks spec	14.	16.47	{0., 35.}	79.6429	0.583428	newly 5 weeks cumul	103.	76.17	{16., 128.}	43.699	0.299265	newly 60 weeks cumul	300.	313.77	{16., 589.}	67.2767	0.438162
45	newly 3 weeks spec	26.	15.44	{0., 37.}	61.0769	0.645672	newly 3 weeks cumul	61.	48.41	{10., 100.}	51.8525	0.349721	newly 5 weeks spec	23.	13.85	{0., 33.}	65.7826	0.833399	newly 5 weeks cumul	105.	76.64	{11., 169.}	54.6667	0.411193	newly 58 weeks cumul	383.	157.68	{11., 337.}	66.5065	0.695881
46	newly 3 weeks spec	13.	5.34	{0., 14.}	74.3077	0.878982	newly 3 weeks cumul	26.	11.45	{2., 25.}	69.1154	0.609117	newly 5 weeks spec	17.	9.61	{0., 26.}	77.1176	0.953577	newly 5 weeks cumul	55.	27.92	{2., 65.}	71.8545	0.666391	newly 65 weeks cumul	489.	418.53	{2., 808.}	63.0695	0.813594
47	newly 3 weeks spec	7.	6.16	{0., 15.}	75.7143	0.579383	newly 3 weeks cumul	19.	14.63	{3., 30.}	61.1053	0.393888	newly 5 weeks spec	6.	8.64	{0., 21.}	125.333	0.737114	newly 5 weeks cumul	28.	30.73	{4., 67.}	80.9643	0.407212	newly 63 weeks cumul	357.	382.78	{4., 808.}	86.4706	0.741819
48	newly 3 weeks spec	17.	25.87	{0., 55.}	106.294	0.637252	newly 3 weeks cumul	40.	85.58	{11., 160.}	138.4	0.387216	newly 5 weeks spec	17.	18.2	{0., 45.}	89.4118	0.880935	newly 5 weeks cumul	72.	125.38	{11., 256.}	109.722	0.396793	newly 58 weeks cumul	326.	254.27	{11., 571.}	60.3834	0.511866
49	newly 3 weeks spec	2.	11.4	{1., 23.}	487.	0.709721	newly 3 weeks cumul	12.	26.23	{7., 49.}	135.25	0.350589	newly 5 weeks spec	8.	17.98	{1., 39.}	163.25	0.539317	newly 5 weeks cumul	27.	59.54	{14., 118.}	142.593	0.372364	newly 62 weeks cumul	311.	543.25	{18., 799.}	104.28	0.453506
50	newly 3 weeks spec	19.	47.64	{31., 63.}	150.842	0.384278	newly 3 weeks cumul	64.	112.74	{80., 146.}	76.5625	0.23657	newly 5 weeks spec	20.	62.22	{40., 82.}	211.1	0.478276	newly 5 weeks cumul	105.	230.05	{162., 288.}	119.095	0.330251	newly 59 weeks cumul	406.	740.67	{647., 810.}	82.431	0.259166
